# Fever Screening and Detection of Febrile Arrivals at an International Airport in Korea: Association among Self-reported Fever, Infrared Thermal Camera Scanning, and Tympanic Temperature

**DOI:** 10.4178/epih/e2014004

**Published:** 2014-05-30

**Authors:** Kyung Sook Cho, Jangho Yoon

**Affiliations:** 1 Health Management and Policy Program, College of Public Health and Human Sciences, Oregon State University, Corvallis, OR, USA; 2 Ministry of Health and Welfare, Sejong, Republic of Korea; 3 Applied Economics Graduate Program, Oregon State University, Corvallis, OR, USA

**Keywords:** Fever, Prevalence, Body temperature, Health records, Quarantine

## Abstract

**OBJECTIVES:**

The purpose of this research was to measure fever prevalence and the effectiveness of a fever screening procedure in detecting febrile arrivals at an international airport in Korea.

**METHODS:**

Data were retrieved from arrivals’ health declaration forms and questionnaires for febrile arrivals at an international airport collected by a national quarantine station during the year 2012. Self-reported health declaration forms were returned by 355,887 arrivals (61% of the total arrivals). Of these, 608 symptomatic arrivals (0.2%) including 6 febrile arrivals were analyzed.

**RESULTS:**

Fever prevalence at an international airport in Korea was 0.002%. Self-reported fever was significantly positively associated with tympanic temperature (*p*<0.001). The difference between the thermal camera temperature (36.83°C) and tympanic (or ear) temperature (38.14°C) was not statistically significant.

**CONCLUSIONS:**

The findings imply that a procedure for mass detection of fever such as self-reported questionnaires and thermal camera scanning may serve as an effective tool for detecting febrile arrivals at quarantine stations. Future research can benefit from looking at the sensitivity, specificity, positive predictive value, and negative predictive value of the entry screening system.

## INTRODUCTION

Global outbreaks of severe acute respiratory syndrome (SARS) in 2003 have led many countries to reinforce the entry screening systems at their international airports, ports, and border lines as a way to prevent or delay the spread of infectious diseases across countries [1-4]. An entry screening often involves infrared thermal scanning for a mass detection of febrile passengers because fever is one of the most noticeable signs of infections such as avian influenza, influenza A virus subtype H1N1, and SARS [5,6]. Despite some controversy [4,7], most prior studies report that the border fever surveillance system significantly identifies febrile arrivals, and is an effective measure of the early detection of febrile passengers [1,2,8,9].

In Korea, during the global SARS outbreak of 2003, thirteen quarantine stations nationwide adopted infrared thermal camera scanning to identify febrile arrivals. Since then, the process for the detection of febrile arrivals has been reinforced as follows. First, arrivals passing through airports, ports, or border lines are inspected by infrared thermal cameras, and also passengers are required to complete and submit health declaration forms, called self-reported health questionnaires. Then, ear (or tympanic) temperature–which has been the protocol since 2010–is checked for individuals who have any self-reported symptoms or those who are detected by infrared thermal scanning. Individuals with a fever above 37.8°C are interviewed by quarantine officers or doctors, and their ear temperature and thermal camera temperature are measured again. Finally, laboratory testing is used on final suspected cases to confirm patients with an infectious disease [10]. The entire procedure therefore relies on the effectiveness of initial fever detection by infrared thermal camera and health declaration forms.

This research represents the first study that reports the prevalence of febrile arrivals at an international airport in Korea. Importantly, we also test an association between fever measurements (e.g., self-reported fever and tympanic temperature) and thermal camera temperature. Prior studies [1,2,8,9] imply that the mass fever screening in quarantine stations in Korea may effectively detect arrivals with fever. However, there is no fever screening-related study for border control or official statistics on fever prevalence in Korea’s quarantine stations. Quarantine-related studies are extremely rare in Korea [11].

## MATERIALS AND METHODS

### Data source and study subjects

**Figure 1.**
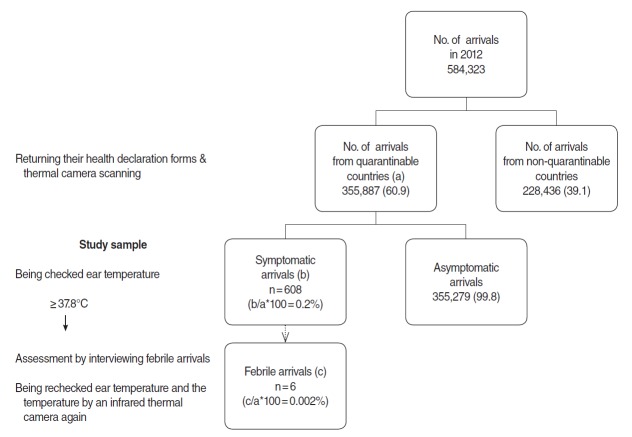
The process that was used to select study participants from the entire arrivals. Values are presented as number (%).

Data were retrieved from health declaration forms, as well as from records of in-person interviews for febrile arrivals at an international airport of a National Quarantine Station in Korea. Figure 1 depicts the process used to select study participants from the entire arrivals. The total of 584,323 arrivals passed through the airport from January 1 to December 31 in 2012. Among those, 355,887 arrivals (61% of the total arrivals) came from quarantinable countries designated by the Korean Minister of Health and Welfare. Passengers from the quarantinable countries must return completed health declaration forms at any international airports, seaports, and border lines at the time of entry into Korea. The final study sample includes 608 subjects (313 females and 294 males) aged 1 to 86 who self-reported at least one symptom of runny nose, stuffy nose, sore throat, cough, and fever.

### Measures

Our measures include dichotomous self-reported fever, thermal camera temperature, and tympanic temperature, which were collected as followings. Arrivals from quarantinable countries filled out health declaration forms distributed by flight attendants, and submitted the completed forms to quarantine officers when passing by infrared thermal cameras (Thermovision A20M; FLIR, Wilsonville, OR, USA; Thermo Tracer TH7800, ThermoGraphy R300; NEC, Tokyo, Japan). The quarantine officers set the thermal cameras for 36°C, and checked the maximum temperature on the camera screens at ringed alarms when passengers walked by the infrared thermal cameras. Symptomatic arrivals were classified as passengers whose health declaration forms self-reported one or more the symptoms or those who were identified by the thermal camera scanning to have a fever above 36°C. There was no case that was an asymptomatic arrival but detected by the thermal camera scanning. Quarantine officers then measures symptomatic arrivals’ tympanic temperature (ThermoScan IRT-3020, ThermoScan IRT-4020: Braun, Kronberg, Germany) and passengers with a fever above 37.8°C were identified as febrile arrivals. Once identified, febrile arrivals were interviewed by quarantine officers or a physician. During the interview, the mean of right and left ear temperatures and thermal camera temperature were measured again. Temperatures reported in this research for febrile arrivals represent the first and second measurements of ear temperatures and the second measurement of thermal camera temperature.

Two quarantine officers retrieved information from the returned health declaration forms, including arrival date, nationality, age, gender, countries of stay during the past 10 days before arrival, and health-related symptoms during the past 10 days before arrival (such as runny or stuffy nose, sore throat, cough, fever, diarrhea, vomiting, abdominal pain, difficulty breathing, and shortness of breath). Tympanic temperatures were retrieved from records for the 608 arrivals detected by health declaration forms and the thermal camera scanning. In addition, second tympanic temperatures and re-measured thermal camera temperatures were retrieved from records for the six febrile arrivals who had a temperature above 37.8°C based on their initial ear temperatures.

### Statistical analysis

A chi-squared test was used to analyze correlation between self-reported fever and tympanic temperature for symptomatic arrivals. Considering the Shapiro-Wilk test revealed that the thermal camera temperature and tympanic temperature were normally distributed (*p*>0.005), we employed the parametric paired *t*-test to assess statistical significance of the difference between thermal camera temperature and tympanic temperature for febrile arrivals. All analyses were done in PASW version 18.0 (SPSS Inc., Chicago, IL, USA).

## RESULTS

### Sample characteristics and fever prevalence

Approximately half of the entire 608 subjects (48.4%) were males (294 cases), shown in Table 1. The mean age was 25.1 years. Teens (37.7%) comprised the most frequent age group, followed by adults aged 20-29 and 30-39. Arrivals in 40s and 50s accounted for 7.1% and 4.6% of the sample, respectively. Only 1.2% of the sample was the elderly over 60 years of age, and 3.8% were under 10 years. More than half of the sample was Chinese (55.9%), 37% Korean, 4.3% other Asian, and 1.6% European. Most of the subjects (96.4%) stayed only in China, and 3.6% stayed other countries in addition to China during the past 10 days.

The fever screening at the international airport identified six febrile arrivals–i.e., those with tympanic temperature above 37.8°C (Figure 1). This implies a fever prevalence of 0.002% (6 cases) among the total 355,887 arrivals from quarantinable countries. Further, the six cases comprised approximately 1% of the 608 symptomatic arrivals: two males and four females. Two cases were in 20s of age, another two in 30s, and two in 50s. Four subjects were Chinese and two were Korean. All six febrile arrivals stayed in China during the past 10 days before arrival (Table 1).

**Figure 2.**
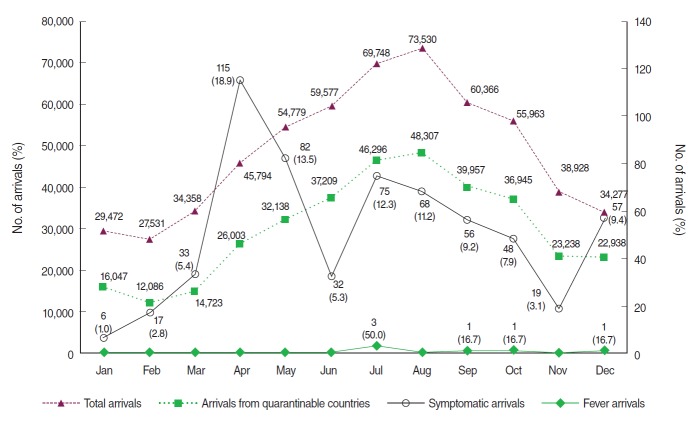
Monthly total arrivals, arrivals from quarantinable countries, symptomatic arrivals and fever arrivals.

The monthly distributions of total arrivals as well as arrivals from quarantinable countries reveal that the arrivals had increased since January, peaked at the midyear 2012, and then reduced gradually (Figure 2). Summer represented a season that had more arrivals than other seasons, followed by fall, spring, and winter. In comparison, symptomatic arrivals were identified the most frequently in April (n=115; 18.9%). July, August, September and December had 12.3%, 11.2%, 9.2%, and 9.4% of the entire symptomatic arrivals, respectively. There were three febrile arrivals in July, and one in each September, October and November.

### Self-reported symptoms and tympanic temperature

Table 1 also presents that the most frequent symptom among symptomatic arrivals was runny or stuffy nose (59.1%). Cough comprised the second most frequent symptom (48.8%). 18.8% of the symptomatic arrivals had sore throat, and 5.1% had abdominal pain and fever. Other symptoms include; diarrhea (2.8%), vomiting (2.0%), shortness of breath (1.3%), and difficulty in breathing (0.7%). 79.1% of the symptomatic arrivals had tympanic temperature under 36.7°C, and 19.9% had tympanic temperature of 36.7°C to 37.7°C.

**Figure 3.**
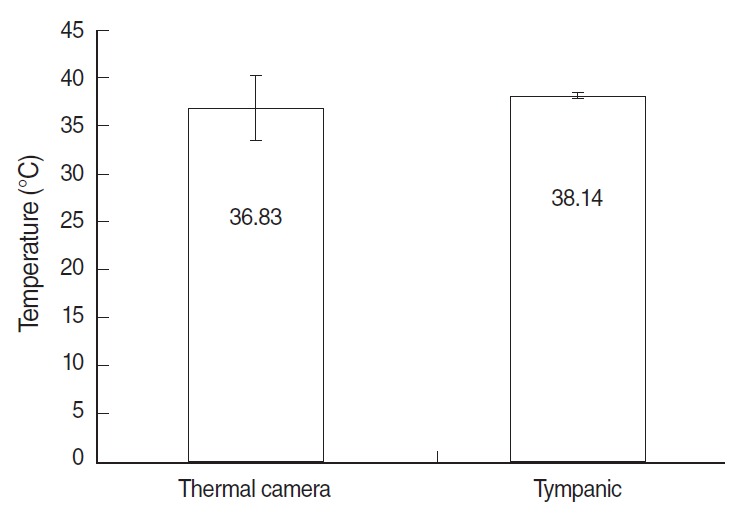
Association between thermal camera temperature and tympanic temperature in febrile arrivals (n=6). Both of them were second measurements and the tympanic temperature was the mean of right and left ear temperatures; p=0.316 (t=-1.114) calculated by paired t-test.

Of the six febrile arrivals, three subjects reported cough, two had sore throats, two had fevers, and one had an abdominal pain. The rechecked tympanic temperature (38.14°C) was similar to the first one (38.20°C), with the thermal temperature (36.83°C) slightly lower than the ear temperature (Table 1 and Figure 3).

### Correlation among measurements for fever

Table 2 presents the association between self-reported fever and tympanic temperature. Among self-reported fever arrivals (31 cases), 2 cases (6.5%) was confirmed as the febrile arrivals with a temperature above 37.8°C. Of all non-self-reported fever arrivals (577 cases), 0.7% (4 cases) were identified as febrile arrivals. The χ^2^-test statistic shows that this discrepancy appears to be different at the 99.9% level of statistical significance. The results on the difference between the thermal camera temperature and tympanic temperature (n=6) reveals that average temperature from thermal camera scanning and average tympanic temperature were 36.83°C and 38.14°C, respectively. As shown in Figure 3, the paired *t*-test did not reject the null hypothesis, indicating that there is no significant difference between thermal camera scanning and tympanic temperatures.

## DISCUSSION

Despite some skepticism [4-7,12], critics often agree that a border fever surveillance system can be useful for early detection of imported infection [1,2,8,9]. A recent study from Korea suggests that a fever over 37.8°C is the most accurate predictor for cases confirmed of pandemic H1N1/09 compared with the cases of influenza-like illness [6]. Similarly, Poutanen et al. [13] reported that all SARS patients in Canada had fevers and Tran et al. [14] documented that all avian influenza A (H5N1) patients in Vietnam had fevers over 38.5°C. These studies collectively imply that entry screening can effectively start with a detection of febrile passengers.

In fact, Korea, along with Japan, has implemented the most rigid entry screening for the detection of febrile arrivals [2,10]. Korea utilizes the self-reported health declaration form and thermal camera scanning to detect symptomatic arrivals. Febrile arrivals are subsequently identified as those with the tympanic temperature of at least 37.8°C [10]. In Taiwan, quarantine officers administer a symptom survey only for arrivals who were identified by the thermal camera scanning to have a temperature of 37.5°C or higher. Febrile arrivals, in turn, are defined as those with ear temperature of 38°C or higher [1]. Australia defines febrile arrivals as those who run a body temperature of 38°C or higher, but does not use the thermal camera scanning. Rather, trained nurses assess clinical symptoms and measure tympanic temperature [3].

This study analyzed data on airline travelers who arrived at an international airport in Korea in 2012. Among 355,887 (61% of the total arrivals) of arrivals who returned their health declaration forms, 608 cases (0.2%) who reported subjective symptoms such as runny rose and fever were analyzed. Febrile arrivals were defined as individuals with above 37.8°C tympanic temperature. This threshold is frequently used elsewhere [6,7, 11,15]. We discovered an annual fever prevalence of 0.002% (6 cases) at an international airport in Korea among passengers from quarantinable countries. The fever prevalence is lower than 0.004% during the influenza pandemic of 2009 in Japan [2] and the 0.08-0.10% of Taiwan for the years 2007-2010 [1], though they may not be directly comparable to one another because fever prevalence varies by influenza season, region and country of origin among arrivals.

Findings show that the proportion of febrile arrivals among self-reported fever arrivals was significantly higher than that of febrile arrivals among non-self-reported fever arrivals. This implies that self-reporting of fever through required health declaration forms can be useful to detect febrile arrivals. Nonetheless, two-thirds of the entire febrile arrivals did not reported fever. In addition, half of the febrile arrivals reported cough that is one of the most important predictors of influenza [11]. Taken together, the findings suggest that quarantine officers be concerned not only about self-reported fever but also about other symptoms such as cough for a detection of imported infection. Korea currently measures tympanic temperature for all cases with any symptoms along with self-reported fever. In so doing, it endeavors to detect febrile arrivals who do not self-report a fever. This study also finds no significant difference between thermal camera temperature and ear temperature. Therefore, an array of the procedures employed by quarantine stations in Korea–health declaration form, thermal camera scanning, and subsequently tympanic temperature measurement–could serve as useful complements to one another in detecting febrile arrivals as accurately as possible.

An important limitation of this study is noteworthy. In this study, we were not able to perform multivariate analysis because of the so-called small cell problem (i.e., only six cases of febrile arrivals). Nonetheless, this research, for the first time in Korea analyzed the fever prevalence and the effectiveness of mass thermal camera scanning to detect fever at an international airport in Korea. Age and outdoor temperature could lead to differential tympanic and thermal camera temperature [16,17]. Future research can add to the literature by teasing out the influence of the factors on the detection of febrile arrivals. Our data do not include arrivals who might have been asymptomatic but detected as having a fever by the thermal camera scanning. Also, we were not able to identify those who self-reported a fever but were not detected as a fever case by the thermal camera scanning. Future research can benefit from having a stronger research design that includes these missed cases in analyses. The limitation also warrants future efforts to measure sensitivity, specificity, positive predictive value and negative predictive value of the thermal camera, self-reported fever or entry screening system.

## Figures and Tables

**Table 1.** Passenger characteristics, symptoms, and tympanic and thermal camera temperature

Variables	Symptomatic arrivals (n = 608)	Febrile arrivals (n = 6)
Gender		
Male	294 (48.4)	2 (33.3)
Female	313 (51.6)	4 (66.7)
Age	25.1 ±12.7	40.3±15.5
≤9	23 (3.8)	0 (0.0)
10-19	229 (37.7)	0 (0.0)
20-29	152 (25.5)	2 (33.3)
30-39	113 (19.0)	2 (33.3)
40-49	43 (7.1)	0 (0.0)
50-59	28 (4.6)	2 (33.4)
≥60	7(1.2)	0 (0.0)
Nationality		
China	340 (55.9)	4 (66.7)
Korea	224 (36.8)	2 (33.3)
Other countries in Asia	26 (4.3)	0 (0.0)
Countries in Europe	10 (1.6)	0 (0.0)
Others	8 (1.3)	0 (0.0)
Stayed countries during past 10 days before arrival		
China	586 (96.4)	6 (100)
China and other countries	22 (3.6)	0 (0.0)
Self-reported symptoms		
Runny or stuffy nose	359 (59.1)	0 (0.0)
Sore throat	114 (18.8)	2 (33.3)
Cough	296 (48.8)	3 (50.0)
Fever	31 (5.1)	2 (33.3)
Diarrhea	17 (2.8)	0 (0.0)
Vomiting	12 (2.0)	0 (0.0)
Abdominal pain	31 (5.1)	1 (16.7)
Difficulty breathing	4 (0.7)	0 (0.0)
Shortness of breath	8 (1.3)	0 (0.0)
Medication	n/a	3 (50.0)
Tympanic temperature	36.37±0.46	38.20±0.44
(1st measurement) (°C)		
<36.7	481 (79.1)	0 (0.0)
36.7-37.7	121 (19.9)	0 (0.0)
≥37.8	6 (1.0)	6 (100.0)

Values are presented as mean±standard deviation or number (%). Total numbers are not consistent because of missing values. n/a, not applicable.

**Table 2.** Association between self-reported fever and tympanic temperature

Tympanic temperature (°C)	Self-reported fever		Total	χ^2^ (p-value)
	Yes	No		
< 36.7	17 (54.8)	464 (80.4)	481 (79.1)	18.1 (<0.001)
36.7-37.7	12 (38.7)	109 (18.9)	121 (19.9)	
≥37.8	2 (6.5)	4 (0.7)	6 (1.0)	
Total	31 (100)	577 (100)	608 (100)	

Values are presented as number (%).
